# A second monoclinic polymorph of (pyridine-2-carboxaldehyde oximato-κ^2^ *N*,*N*′)(pyridine-2-carboxaldehyde oxime-κ^2^ *N*,*N*′)palladium(II) chloride

**DOI:** 10.1107/S1600536812001559

**Published:** 2012-01-21

**Authors:** Kwang Ha

**Affiliations:** a School of Applied Chemical Engineering, The Research Institute of Catalysis, Chonnam National University, Gwangju 500-757, Republic of Korea

## Abstract

The asymmetric unit of the title compound, [Pd(C_6_H_5_N_2_O)(C_6_H_6_N_2_O)]Cl, contains one half of a cationic Pd^II^ complex and a Cl^−^ anion, with a crystallographic mirror plane parallel to the *ac* plane passing through the Pd and Cl atoms. In the complex, the Pd^II^ ion is four-coordinated in a distorted square-planar environment by four N atoms derived from the two chelating ligands. The hy­droxy H atom lies on the mirror plane and so is equidistant from the O atoms. This indicates that the negative charge is delocalized over the two O atoms. The complex mol­ecules are stacked in columns along the *c* axis and are connected by C—H⋯O hydrogen bonds, forming a three-dimensional network. The structure reported herein represents a new monoclinic polymorph of the previously reported monoclinic (*C*2/*c*) form [Torabi *et al.* (2007 ▶). *Z. Kristallogr. New Cryst. Struct*. **222**, 197–198].

## Related literature

For the *C*2/*c* polymorph of the title compound, see: Torabi *et al.* (2007 ▶). For the crystal structure of the related complex [PdCl_2_(C_6_H_6_N_2_O)], see: Ha (2011 ▶).
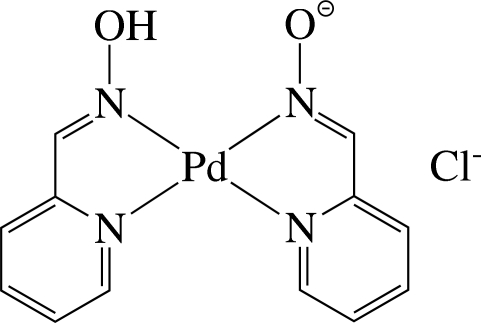


## Experimental

### 

#### Crystal data

[Pd(C_6_H_5_N_2_O)(C_6_H_6_N_2_O)]Cl
*M* *_r_* = 385.10
Monoclinic, 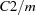

*a* = 14.0865 (16) Å
*b* = 12.1439 (14) Å
*c* = 8.2723 (9) Å
β = 114.447 (2)°
*V* = 1288.2 (3) Å^3^
*Z* = 4
Mo *K*α radiation
μ = 1.65 mm^−1^
*T* = 200 K
0.22 × 0.15 × 0.10 mm

#### Data collection

Bruker SMART 1000 CCD diffractometer
Absorption correction: multi-scan (*SADABS*; Bruker, 2000 ▶) *T*_min_ = 0.894, *T*_max_ = 1.000
3935 measured reflections
1312 independent reflections
1188 reflections with *I* > 2σ(*I*)
*R*_int_ = 0.025

#### Refinement

*R*[*F* ^2^ > 2σ(*F* ^2^)] = 0.030
*wR*(*F* ^2^) = 0.075
*S* = 1.09
1312 reflections
97 parameters
H atoms treated by a mixture of independent and constrained refinement
Δρ_max_ = 0.75 e Å^−3^
Δρ_min_ = −0.53 e Å^−3^

### 

Data collection: *SMART* (Bruker, 2000 ▶); cell refinement: *SAINT* (Bruker, 2000 ▶); data reduction: *SAINT*; program(s) used to solve structure: *SHELXS97* (Sheldrick, 2008 ▶); program(s) used to refine structure: *SHELXL97* (Sheldrick, 2008 ▶); molecular graphics: *ORTEP-3* (Farrugia, 1997 ▶) and *PLATON* (Spek, 2009 ▶); software used to prepare material for publication: *SHELXL97*.

## Supplementary Material

Crystal structure: contains datablock(s) global, I. DOI: 10.1107/S1600536812001559/tk5047sup1.cif

Structure factors: contains datablock(s) I. DOI: 10.1107/S1600536812001559/tk5047Isup2.hkl

Additional supplementary materials:  crystallographic information; 3D view; checkCIF report

## Figures and Tables

**Table:** 

Pd1—N1	2.068 (3)
Pd1—N2	1.985 (3)

**Table:** 

N2—Pd1—N1	78.91 (14)

**Table 2:** Hydrogen-bond geometry (Å, °)

*D*—H⋯*A*	*D*—H	H⋯*A*	*D*⋯*A*	*D*—H⋯*A*
O1—H1*o*⋯O1^i^	1.21 (1)	1.21 (1)	2.418 (7)	175 (7)
C2—H2⋯O1^ii^	0.95	2.49	3.178 (5)	129

Symmetry codes: (i) 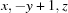; (ii) 
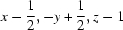
.

**Fig. 1.**
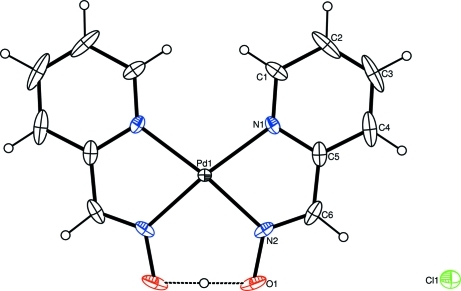
A view of the molecular structure of the title compound, with displacement ellipsoids drawn at the 30% probability level. Unlabelled atoms are related to the reference atoms by the (x, 1-y, z) symmetry transformation.

**Fig. 2.**
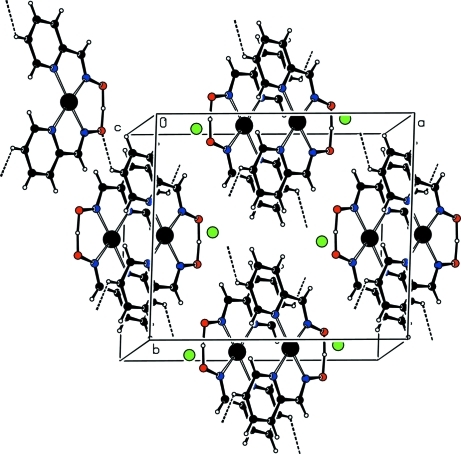
A view of the crystal packing of the title compound. Intermolecular hydrogen-bond interactions are drawn with dashed lines.

## supplementary crystallographic information

### Comment

The title compound, [Pd(C_12_H_11_N_4_O_2_)]Cl, was obtained as a by-product from the reaction of Na_2_PdCl_4_ with *syn*-2-pyridinealdoxime in H_2_O. The X-ray crystal structure of the compound was previously reported in the monoclinic space group *C*2/*c* (Torabi *et al.*, 2007). In the latter, the molecule does not exhibit molecular symmetry. The structure presented herein is essentially the same as the published structure but represents a new monoclinic polymorph with the space group *C*2/*m*. The yellow main product of the reaction, [PdCl_2_(C_6_H_6_N_2_O)], was investigated previously (Ha, 2011).

The asymmetric unit of the title compound contains one half of a cationic Pd^II^ complex and a Cl^-^ anion (Fig. 1). The compound is disposed about a crystallographic mirror plane parallel to the *ac* plane passing through the Pd and Cl atoms. In the complex, the Pd^II^ ion is four-coordinated in a distorted square-planar environment by four N atoms of the two chelating ligands. Formally, one of the ligands is coordinated to the Pd atom in the monoanionic form, but the negative charge is delocalized over the two O atoms of the ligands; the hydroxy H atom is located in the middle of the O atoms forming a nearly planar six-membered ring. The tight N1—Pd1—N2 chelate angle of 78.91 (14)° contributes the distortion of the square planar structure. The *trans* N1—Pd1—N2^i^ (symmetry code i: *x*,1 - *y*, *z*) bond angle is 173.62 (12)°. The Pd1—N1(pyridine) bond length is slightly longer than the Pd1—N2(oxime) bond length (Table 1). The ligands are nearly planar, with a maximum deviation of 0.024 (3) Å from the least-squares plane, and the dihedral angle between the ligands is 5.06 (8)°. The complex molecules are stacked in columns along the *c* axis and are connected by intermolecular C—H···O hydrogen bonds, forming a three-dimensional network (Fig. 2 and Table 2). In the columns, intermolecular π-π interactions between the pyridine rings are present, the shortest ring centroid-centroid distance being 3.787 (3) Å.

### Experimental

The title compound was obtained as a by-product from the reaction of Na_2_PdCl_4_ (0.2942 g, 1.000 mmol) with *syn*-2-pyridinealdoxime (0.2444 g, 2.001 mmol) in H_2_O (20 ml). After stirring of the reaction mixture for 3 h at room temperature, the formed precipitate was separated by filtration, washed with H_2_O and acetone, to give the main product as a yellow powder (0.2302 g) (Ha, 2011). The orange by-product in a small amount was obtained from the mixture of filtrate and washing solution. Crystals suitable for X-ray analysis were obtained by slow evaporation from an *N,N*-dimethylformamide (DMF) solution of the by-product at 60 °C.

### Refinement

H atoms were positioned geometrically and allowed to ride on their respective parent atoms [C—H = 0.95 Å and *U*_iso_(H) = 1.2*U*_eq_(C)]. The hydroxy H atom was located from Fourier difference maps and refined isotropically. A number of outlying reflections, *i.e*. (6 0 7), (9 7 4), (11 9 7), (12 2 9), (12 4 9), (10 8 8), (17 1 6), (8 8 8), (10 0 10), (12 0 9), (6 14 1), (11 5 9) and (0 2 4), were omitted owing to poor agreement.

### Crystal data

**Table:**

[Pd(C_6_H_5_N_2_O)(C_6_H_6_N_2_O)]Cl	*F*(000) = 760
*M**_r_* = 385.10	*D*_x_ = 1.986 Mg m^−^^3^
Monoclinic, *C*2/*m*	Mo *K*α radiation, λ = 0.71073 Å
Hall symbol: -C 2y	Cell parameters from 2872 reflections
*a* = 14.0865 (16) Å	θ = 2.3–26.0°
*b* = 12.1439 (14) Å	µ = 1.65 mm^−^^1^
*c* = 8.2723 (9) Å	*T* = 200 K
β = 114.447 (2)°	Prism, orange
*V* = 1288.2 (3) Å^3^	0.22 × 0.15 × 0.10 mm
*Z* = 4	

### Data collection

**Table:**

Bruker SMART 1000 CCD diffractometer	1312 independent reflections
Radiation source: fine-focus sealed tube	1188 reflections with *I* > 2σ(*I*)
graphite	*R*_int_ = 0.025
φ and ω scans	θ_max_ = 26.0°, θ_min_ = 2.3°
Absorption correction: multi-scan (*SADABS*; Bruker, 2000)	*h* = −17→17
*T*_min_ = 0.894, *T*_max_ = 1.000	*k* = −12→14
3935 measured reflections	*l* = −10→10

### Refinement

**Table:**

Refinement on *F*^2^	Primary atom site location: structure-invariant direct methods
Least-squares matrix: full	Secondary atom site location: difference Fourier map
*R*[*F*^2^ > 2σ(*F*^2^)] = 0.030	Hydrogen site location: inferred from neighbouring sites
*wR*(*F*^2^) = 0.075	H atoms treated by a mixture of independent and constrained refinement
*S* = 1.09	*w* = 1/[σ^2^(*F*_o_^2^) + (0.035*P*)^2^ + 2.4037*P*] where *P* = (*F*_o_^2^ + 2*F*_c_^2^)/3
1312 reflections	(Δ/σ)_max_ < 0.001
97 parameters	Δρ_max_ = 0.75 e Å^−^^3^
0 restraints	Δρ_min_ = −0.53 e Å^−^^3^

### Special details

**Table:**

Geometry. All e.s.d.'s (except the e.s.d. in the dihedral angle between two l.s. planes) are estimated using the full covariance matrix. The cell e.s.d.'s are taken into account individually in the estimation of e.s.d.'s in distances, angles and torsion angles; correlations between e.s.d.'s in cell parameters are only used when they are defined by crystal symmetry. An approximate (isotropic) treatment of cell e.s.d.'s is used for estimating e.s.d.'s involving l.s. planes.
Refinement. Refinement of *F*^2^ against ALL reflections. The weighted *R*-factor *wR* and goodness of fit *S* are based on *F*^2^, conventional *R*-factors *R* are based on *F*, with *F* set to zero for negative *F*^2^. The threshold expression of *F*^2^ > σ(*F*^2^) is used only for calculating *R*-factors(gt) *etc*. and is not relevant to the choice of reflections for refinement. *R*-factors based on *F*^2^ are statistically about twice as large as those based on *F*, and *R*- factors based on ALL data will be even larger.

### Fractional atomic coordinates and isotropic or equivalent isotropic displacement parameters (Å^2^)

**Table:**

	*x*	*y*	*z*	*U*_iso_*/*U*_eq_	
Pd1	0.09319 (3)	0.5000	0.41451 (4)	0.02259 (15)	
O1	0.2571 (2)	0.4005 (3)	0.7251 (4)	0.0546 (9)	
H1o	0.261 (5)	0.5000	0.725 (10)	0.08 (2)*	
N1	0.0142 (2)	0.3633 (2)	0.2741 (4)	0.0295 (7)	
N2	0.1746 (2)	0.3793 (3)	0.5732 (4)	0.0342 (7)	
C1	−0.0700 (3)	0.3564 (4)	0.1201 (5)	0.0444 (11)	
H1	−0.1006	0.4219	0.0576	0.053*	
C2	−0.1134 (4)	0.2558 (6)	0.0502 (8)	0.075 (2)	
H2	−0.1730	0.2539	−0.0599	0.090*	
C3	−0.0738 (6)	0.1612 (5)	0.1328 (11)	0.092 (3)	
H3	−0.1051	0.0926	0.0848	0.110*	
C4	0.0139 (5)	0.1668 (4)	0.2904 (9)	0.0721 (18)	
H4	0.0452	0.1013	0.3524	0.086*	
C5	0.0562 (3)	0.2684 (3)	0.3576 (6)	0.0413 (10)	
C6	0.1470 (3)	0.2806 (4)	0.5255 (6)	0.0457 (11)	
H6	0.1831	0.2191	0.5946	0.055*	
Cl1	0.23809 (11)	0.0000	0.72959 (19)	0.0424 (3)	

### Atomic displacement parameters (Å^2^)

**Table:**

	*U*^11^	*U*^22^	*U*^33^	*U*^12^	*U*^13^	*U*^23^
Pd1	0.0211 (2)	0.0221 (2)	0.0211 (2)	0.000	0.00530 (15)	0.000
O1	0.0321 (15)	0.080 (2)	0.0384 (16)	0.0115 (16)	0.0015 (13)	0.0273 (17)
N1	0.0320 (15)	0.0266 (17)	0.0349 (17)	−0.0057 (13)	0.0187 (14)	−0.0088 (13)
N2	0.0270 (15)	0.044 (2)	0.0316 (17)	0.0108 (14)	0.0122 (13)	0.0164 (15)
C1	0.036 (2)	0.058 (3)	0.042 (2)	−0.018 (2)	0.0183 (19)	−0.023 (2)
C2	0.066 (3)	0.100 (5)	0.078 (4)	−0.058 (4)	0.048 (3)	−0.066 (4)
C3	0.120 (6)	0.059 (4)	0.141 (6)	−0.064 (4)	0.098 (5)	−0.069 (4)
C4	0.109 (4)	0.025 (2)	0.127 (5)	−0.012 (3)	0.093 (4)	−0.015 (3)
C5	0.051 (2)	0.027 (2)	0.066 (3)	−0.0016 (18)	0.044 (2)	−0.002 (2)
C6	0.048 (2)	0.037 (2)	0.068 (3)	0.020 (2)	0.040 (2)	0.027 (2)
Cl1	0.0381 (7)	0.0499 (9)	0.0418 (8)	0.000	0.0190 (6)	0.000

### Geometric parameters (Å, °)

**Table:**

Pd1—N1	2.068 (3)	C1—H1	0.9500
Pd1—N1^i^	2.068 (3)	C2—C3	1.335 (10)
Pd1—N2^i^	1.985 (3)	C2—H2	0.9500
Pd1—N2	1.985 (3)	C3—C4	1.377 (9)
O1—N2	1.337 (4)	C3—H3	0.9500
O1—H1o	1.210 (5)	C4—C5	1.383 (6)
N1—C1	1.336 (5)	C4—H4	0.9500
N1—C5	1.347 (5)	C5—C6	1.455 (6)
N2—C6	1.271 (5)	C6—H6	0.9500
C1—C2	1.381 (7)		
			
N2^i^—Pd1—N2	95.2 (2)	C3—C2—C1	121.9 (6)
N2^i^—Pd1—N1	173.62 (12)	C3—C2—H2	119.1
N2—Pd1—N1	78.91 (14)	C1—C2—H2	119.1
N2^i^—Pd1—N1^i^	78.91 (14)	C2—C3—C4	117.6 (5)
N2—Pd1—N1^i^	173.62 (13)	C2—C3—H3	121.2
N1—Pd1—N1^i^	106.87 (17)	C4—C3—H3	121.2
N2—O1—H1o	103 (3)	C3—C4—C5	119.5 (5)
C1—N1—C5	117.6 (4)	C3—C4—H4	120.2
C1—N1—Pd1	130.1 (3)	C5—C4—H4	120.2
C5—N1—Pd1	112.3 (3)	N1—C5—C4	122.2 (5)
C6—N2—O1	120.6 (3)	N1—C5—C6	115.3 (3)
C6—N2—Pd1	118.2 (3)	C4—C5—C6	122.5 (5)
O1—N2—Pd1	121.3 (3)	N2—C6—C5	115.3 (4)
N1—C1—C2	121.3 (5)	N2—C6—H6	122.3
N1—C1—H1	119.4	C5—C6—H6	122.3
C2—C1—H1	119.4		
			
N2—Pd1—N1—C1	178.8 (3)	C2—C3—C4—C5	1.1 (8)
N1^i^—Pd1—N1—C1	1.6 (4)	C1—N1—C5—C4	−1.1 (5)
N2—Pd1—N1—C5	0.0 (2)	Pd1—N1—C5—C4	177.9 (3)
N1^i^—Pd1—N1—C5	−177.22 (17)	C1—N1—C5—C6	−179.4 (3)
N2^i^—Pd1—N2—C6	178.0 (2)	Pd1—N1—C5—C6	−0.4 (4)
N1—Pd1—N2—C6	0.4 (3)	C3—C4—C5—N1	0.1 (7)
N2^i^—Pd1—N2—O1	−2.8 (3)	C3—C4—C5—C6	178.3 (4)
N1—Pd1—N2—O1	179.6 (3)	O1—N2—C6—C5	−180.0 (3)
C5—N1—C1—C2	0.9 (5)	Pd1—N2—C6—C5	−0.7 (5)
Pd1—N1—C1—C2	−177.9 (3)	N1—C5—C6—N2	0.7 (5)
N1—C1—C2—C3	0.3 (7)	C4—C5—C6—N2	−177.6 (4)
C1—C2—C3—C4	−1.3 (8)		

Symmetry codes: (i) *x*, −*y*+1, *z*.

### Hydrogen-bond geometry (Å, °)

**Table:**

*D*—H···*A*	*D*—H	H···*A*	*D*···*A*	*D*—H···*A*
O1—H1o···O1^i^	1.21 (1)	1.21 (1)	2.418 (7)	175 (7)
C2—H2···O1^ii^	0.95	2.49	3.178 (5)	129

Symmetry codes: (i) *x*, −*y*+1, *z*; (ii) *x*−1/2, −*y*+1/2, *z*−1.
